# Three-Year Change in the Wellbeing of Orphaned and Separated Children in Institutional and Family-Based Care Settings in Five Low- and Middle-Income Countries

**DOI:** 10.1371/journal.pone.0104872

**Published:** 2014-08-27

**Authors:** Kathryn Whetten, Jan Ostermann, Brian W. Pence, Rachel A. Whetten, Lynne C. Messer, Sumedha Ariely, Karen O'Donnell, Augustine I. Wasonga, Vanroth Vann, Dafrosa Itemba, Misganaw Eticha, Ira Madan, Nathan M. Thielman, The Positive Outcomes for Orphans (POFO) Research Team

**Affiliations:** 1 Center for Health Policy, Duke Global Health Institute, Duke University, Durham, North Carolina, United States of America; 2 Terry Sanford Institute of Public Policy, Duke University, Durham, North Carolina, United States of America; 3 Department of Epidemiology, School of Public Health, University of North Carolina at Chapel Hill, Chapel Hill, North Carolina, United States of America; 4 School of Community Health, College of Urban and Public Affairs, Portland State University, Portland, Oregon, United States of America; 5 Departments of Psychiatry and Pediatrics, Duke University Medical Center, Durham, North Carolina, United States of America; 6 Center for Child and Family Health, Duke University, Durham, North Carolina, United States of America; 7 ACE Africa, Bungoma, Kenya; 8 Homeland, Battambang, Cambodia; 9 TAWREF, Moshi, Tanzania; 10 Stand for Vulnerable Organization, Addis Ababa, Ethiopia; 11 Sahara House, Delhi, India; 12 Department of Medicine, Division of Infectious Diseases and International Health, Duke University, Durham, North Carolina, United States of America; University of Southampton, United Kingdom

## Abstract

**Background:**

With more than 2 million children living in group homes, or “institutions”, worldwide, the extent to which institution-based caregiving negatively affects development and wellbeing is a central question for international policymakers.

**Methods:**

A two-stage random sampling methodology identified community representative samples of 1,357 institution-dwelling orphaned and separated children (OSC) and 1,480 family-dwelling OSC aged 6–12 from 5 low and middle income countries. Data were collected from children and their primary caregivers. Survey-analytic techniques and linear mixed effects models describe child wellbeing collected at baseline and at 36 months, including physical and emotional health, growth, cognitive development and memory, and the variation in outcomes between children, care settings, and study sites.

**Findings:**

At 36-month follow-up, institution-dwelling OSC had statistically significantly higher height-for-age Z-scores and better caregiver-reported physical health; family-dwelling OSC had fewer caregiver-reported emotional difficulties. There were no statistically significant differences between the two groups on other measures. At both baseline and follow-up, the magnitude of the differences between the institution- and family-dwelling groups was small. Relatively little variation in outcomes was attributable to differences between sites (11–27% of total variation) or care settings within sites (8–14%), with most variation attributable to differences between children within settings (60–75%). The percent of variation in outcomes attributable to the care setting type, institution- versus family-based care, ranged from 0–4% at baseline, 0–3% at 36-month follow-up, and 0–4% for changes between baseline and 36 months.

**Interpretation:**

These findings contradict the hypothesis that group home placement universally adversely affects child wellbeing. Without substantial improvements in and support for family settings, the removal of institutions, broadly defined, would not significantly improve child wellbeing and could worsen outcomes of children who are moved from a setting where they are doing relatively well to a more deprived setting.

## Introduction

Low and middle income countries (LMICs) [1] are home to an estimated 132 million single and double orphans, 95% of whom are over the age of five [2]. Additionally, tens of millions of street children are in need of care, and their numbers are increasing in many countries. [3] More than two million children are estimated to live in “institutions” [4], a term used broadly in policy and program planning documents to define group homes where children from multiple families live with biologically non-related caregivers. The variability of these group homes is remarkable, ranging from small residences with long-term live-in caregivers, to large facilities with multiple family-like units, to very large facilities with caregivers working in shifts and without assigned responsibility for individual children [5]–[7]. Other large facilities have children assigned to particular caregivers who spend the night and significant amounts of other time in smaller family-like units within the larger structure. Some institutions provide on-site education, while others send their children to local public schools [7]. Similarly, some institutions provide on-site health care services, while others send children to local health facilities for basic or emergency care. Some have high caregiver-to-child ratios and others do not. Some are established only for younger children while others do not accept children under a certain age. Some specifically target their care to HIV-infected children while others exclude those who were diagnosed with HIV. Despite this profound heterogeneity, analyses of the effect of institution-based residence on child wellbeing have largely focused on one specific type of care-setting: infants cared for in large hospital-style institutions that employ shift workers [8]–[12].

The extent to which care in institutions negatively affects children's development and wellbeing has become a central question for international aid policy affecting LMICs with large numbers and rates of children living in adversity [13], [14]. Policy documents and legislation related to the care of children in institutions frequently cite studies of children who as infants lived in socially and emotionally deprived institutions in Romania and Russia. These studies demonstrated powerful negative effects on the infant brain and child development [9]–[11], [15]. When infants were removed from this environment to live with trained, paid, and supervised foster parents in Romania, brain and child development improvements were observed [11], [12].

However, studies that include data from a broader array of cultural and situational contexts find more nuanced results. For example, in a meta-analysis of studies examining children in institutional and family care it was found that while intelligence quotient values trended toward being lower among infants in institutions [9], this relationship was not observed in lower income countries like Ethiopia [16], Kenya [17], and Eritrea [18], and was not observed among children over age five [9]. Importantly, 95% of all orphaned children are over the age of five [2]. Recent assessments of children's nutritional status in Kenya [19] and of psychosocial status in China [20] found children in group homes were doing somewhat better than their family-based comparisons. Another recent study of approximately 1,400 community children and 1,500 group home children in Kenya found that those living in group homes were significantly more likely to have their basic material needs met than those in family care [7]. The authors' own baseline study conducted in six sites across five countries also found that children in institutional settings scored as well as or better than those in family-based settings across a number of measures of physical and emotional wellbeing (see below) [5].

The Positive Outcomes for Orphans (POFO) study is the only study known to the authors that enrolled a statistically representative sample of both institution-based and family-based orphaned and separated children (OSC) from culturally diverse sites across multiple LMICs and is thus positioned to provide robust comparison of the wellbeing of institution-based and family-based OSC [5], [21]–[24]. For the purposes of the study, OSC were defined as children who were single or double orphans or who were separated from their biological parents with no expectation of either parent returning and no contact information for either parent. Between 2006 and 2008, the POFO study randomly selected institutions from a comprehensive institution list developed at the local level and compared the wellbeing of children in those residences to a randomly selected sample of OSC living in family settings in the same areas. Cross-sectional analyses of baseline data found that health, emotional functioning, learning ability, memory, and physical growth were comparable, on average, for the 1,357 children living in 83 institutional care settings and the 1,480 children living with families in 311 community clusters (geographically bounded sampling areas) in the same regions. After adjusting for site, child age, and child gender, there was far greater variability in wellbeing *between individual children* than *between* institutions as a whole versus family settings as a whole, and the distributions of wellbeing measures across children in institutions and family settings were remarkably similar [5].

Taken together, these studies do not support the notion that all institutional settings adversely affect child wellbeing at all ages. This issue is of critical importance because influential global policies and frameworks are being proposed and enacted based on the premise that time spent in any kind of institutional setting at any age damages children and that any family setting is better than any institutional setting [13], [14]. The aim of the current paper is to examine the wellbeing of children living in these settings over time and specifically to test the hypothesis that over a 3 year period, OSC living in institutions would look worse than those living in families across measures of general health, physical health, learning ability, emotional difficulties and memory. This directional hypothesis stems from the policy assumption that institutions are universally negative for child wellbeing and that even if significant negative outcomes from institutional living are not seen at one point in time, they will emerge over the long term. If this assumption is true, changes in wellbeing measures over time should be principally related to the caregiving structure: i.e., institutions vs. family based settings rather than to differences between geographical settings or individual institutions. We test this hypothesis using the three-year longitudinal follow-up data from the POFO cohort.

## Materials and Methods

### Study Description

POFO is an ongoing longitudinal study following a cohort of children, starting at ages 6 to 12, living in institutional or family-based settings in six sites in five low and middle income countries: Battambang District, Cambodia; Addis Ababa, Ethiopia; Bungoma District, Kenya; Nagaland and Hyderabad, India; and Kilimanjaro Region, Tanzania. Children were enrolled between 2006 and 2008 and followed biannually, with varying amounts of information collected on children in different rounds. This analysis used the baseline and 3 year (interquartile range 2.7 to 3.2 years) follow-up data to examine cross-sectional differences and differential changes between OSC living in institutions and family settings. At follow-up the children were ages 8 to 16. The principal measures of child wellbeing, which were collected at baseline and the 3-year follow-up assessment, are: physical growth, general health, emotional difficulties, learning ability, and memory.

### Study Sample

In describing the study sampling strategies the term *site* is used to describe the 6 geographically and culturally distinct areas where the study was conducted; *setting* is used to refer to either institution or family-based living situation; *clusters* are the smaller geographical units within each site, equivalent to a census tract or zip code in the United States, that were randomly selected to identify OSC living in families; and *sampling unit* is used to describe the second level of sampling (i.e., 83 institutions and 311 clusters yielded 394 sampling units). The full sampling strategy and characteristics of the sample have been reported elsewhere [5]. In brief, within the six study sites, the POFO study utilized a two-stage random sampling methodology to identify a representative sample of 1,357 OSC living in institutional settings and 1,480 OSC living in family-based settings.

To sample children in family-based settings, geographic or administrative boundaries were used to define sampling areas (clusters) within each site, from which 50 clusters were randomly selected in each site, and up to five eligible children ages 6–12 years were randomly selected from each cluster. Eligible children were orphans, defined as children for whom one or both parents had died, and separated children, defined as children who had been separated from their parents with no expectation of return and no contact information for either parent. Eligible children were randomly selected from available lists or through a house-to-house census. One child per household was selected to participate in the study. For households with multiple age-eligible children, the child whose name started with the earliest letter in the alphabet was selected to participate.

To sample children from institutional settings, defined as having at least five children from at least two different biological families not related to the caregivers and not in a family home, the research team developed a comprehensive list of all institutions in the region and randomly selected up to 20 institutions per site. In 2 sites with fewer than 20 institutions, all institutions were included. In total, 83 institutions were included in the study. Institutions provided lists of all children aged 6 to 12. Institutions were approached sequentially until 250 children were enrolled, with up to 20 children randomly selected from each institution. If fewer than 20 age–eligible children lived in the institution, all children from that institution were selected.

#### Selection of Caregivers for inclusion in the surveys

The children's (self-identified) primary caregivers were asked to respond to surveys about themselves and the children. In total, 193 institutional caregivers, ranging from 16 institutional caregivers in Nagaland to 52 in Cambodia, and 1,480 community-based caregivers participated in the assessments.

#### Interviewer Training

One local male and female interviewer and a lead investigator from each site were trained on study protocol and procedures. A week-long training took place at a central location with all interviewers and primary investigators present. Following the training, the interviewers continued practicing and were certified only after repeated direct observation or video taping of interviews with local non-study children. The psychological testing was reviewed by the Duke child psychologist for fidelity to standard test procedures. Site visits, with interviewer observation, were conducted during the data collection to further ensure accuracy and consistency across interviewers and sites.

### Data Collection

Written and verbal consent was obtained from all children's primary caregivers and assent was obtained from each participating child. Ethical approval was received from the Institutional Review Boards (IRB) at Duke University and at each of the study sites. Caregiver consent and child assent was recorded on the IRB-approved consent forms. Informed consent procedures and measures are described in detail in previous publications [5], [21]–[24]. Data were collected from the enrolled children and their primary caregivers at baseline and twice annually for three years. Interviews were conducted in the child's residence and all interviews were conducted verbally in the respondent's native language.

### Wellbeing Measures

All questions and measures were reviewed and tested by each site's research team and community advisory board, in collaboration with the US based research team. Survey translations and back-translations were carefully examined and wording modified when necessary to ensure local relevance and the meaning that was intended in the original English questions. Questions were chosen that had been utilized in various countries, cultures, in low-income settings and with participants with varying educational background.

#### Physical Health and Growth

Caregiver-reported child health measures included symptoms of fever, cough, and diarrhea in the last 2 weeks and the general health of the child. The latter was rated on a scale of 1 to 5, from “very poor” to “very good” from the Medical Outcomes Study Short Form 36 [23], [25]. Growth measures included child height and weight. Body Mass Index (BMI) and child height were age- and gender-standardized according to WHO growth charts [26] and reported as “height-for-age” Z-scores and “BMI-for-age” Z-scores.

#### Emotional Wellbeing

The Strengths and Difficulties Questionnaire (SDQ) [27], [28] was asked of children over age 10 (SDQ-self report) and of the caregivers for all children (SDQ-caregiver report). The four difficulties scales (i.e., emotional symptoms, conduct problems, hyperactivity/inattention, peer relationship), each containing 5 questions with responses ranging from 0 to 2, were used to create a summary score ranging from 0 to 40, with higher values signifying more difficulties [27], [28]. The SDQ was chosen for its brevity, its psychometric properties, and its frequent use in studies of children in countries around the globe [29]–[32].

#### Learning ability and Memory

Three subtests from the Kaufman Assessment Battery for Children-II (KABC-II) [33] were used to evaluate learning ability: sequential processing and short term memory; spatial relations and visual motor integration; and visual problem solving abilities were assessed using the Hand Movements, Triangles, and Pattern Reasoning subtests. The KABC-II was chosen to examine learning ability because, due to its structure and theoretical base, it had been successfully utilized in low-resource and culturally diverse settings [33]. Comparisons of the KABC and the *Wechsler Intelligence Scale for Children* (WISC III) indicated that the KABC, with its low verbal demands, is the preferred cognitive assessment across varied cultural settings [34]. Prior POFO analyses of the use of the KABC-II and the “market list” (see below) demonstrated that these measures can be successfully employed with fidelity in non-standard settings in LMICs [21]. The scores reported here are the mean subtest scaled scores using the test's normative data for child age; results range from 0–19 with higher being better.

The Market List, an adaptation and abbreviation of the *California Verbal Learning Test-Children's Version* (CVLT-C) [35], was used to measure children's executive functioning (attention and motivation) as well as verbal learning and memory. Requiring children to encode, store, and retrieve information, the CVLT-C measures multiple aspects of verbal learning and memory recall and is widely used in assessment of memory, learning, and executive functioning, all of which represent foundation skills for complex learning typically encountered in a formal educational setting. With the assistance of the interviewers in each of the five sites, new market lists were adapted to reflect what children would see in their local market. The categories used in the original CVLT-C (things a child would eat, wear, and play with) were maintained to maximize consistency with the original test. For this report, the score used for analysis was the mean of three administrations of the list.

### Analysis

To assess selection bias from loss to follow-up, we first compared the characteristics of children retained vs. lost at 36 months. All subsequent analyses presented here, both of baseline and follow-up measures, were restricted to children retained at 36 months. Statistical analyses of changes in outcomes over 3 years parallel the analyses of baseline data published previously [5]. In brief, standard survey analytic techniques were used to estimate mean values of each outcome at baseline and 36 months, and the change in each outcome from baseline to 36 months post-enrollment, as well as differences between mean levels and changes for institution-living and family-living OSC. Estimates accounted for unequal selection probabilities and the multilevel study design as previously described. Here, sampling weights were multiplied by inverse probability of observation weights (based on age, sex, care setting type, and site) to account for differential loss to follow-up (Table 1).

**Table 1:** Sample characteristics at baseline, among those retained at 36 months and those lost to follow-up.

	Institution-based			Family-based		
	Retained	Lost		Retained	Lost	
	Mean (SD) or N (%)^1^	Mean (SD) or N (%)^2^		Mean (SD) or N (%)^1^	Mean (SD) or N (%)^2^	
Age at baseline (years)	8.8 (1.8)	9.3 (1.8)	<0.001	8.8 (1.8)	9.1 (12.6)	0.018
Male	570 (57.4%)	192 (52.9%)	0.138	684 (53.0%)	94 (49.5%)	0.360
Female	423 (42.6%)	171 (47.1%)		606 (47.0%)	96 (50.5%)	
**Positive outcomes (higher score is better)**						
Caregiver-rated child health (1 to 5)	4.0 (0.7)	4.0 (0.8)	0.751	3.7 (0.8)	3.6 (6.3)	0.159
Height for age Z score (1 unit = 1 SD))	−1.0 (1.4)	−1.0 (1.5)	0.724	−1.0 (1.3)	−0.9 (6.2)	0.324
BMI for age Z score (1 unit = 1 SD)	−0.7 (1.0)	−0.6 (0.9)	0.322	−0.7 (1.2)	−0.9 (53.2)	0.196
Learning ability (K-ABC II, range 0–19)	4.6 (1.9)	4.9 (1.9)	0.075	4.5 (1.8)	4.2 (46.8)	0.054
Memory CVLT (Market list; range 0–15)	7.7 (2.3)	7.9 (2.4)	0.146	7.2 (2.2)	7.2 (90.5)	0.863
**Negative outcomes (higher score or percentage is worse)**						
Emotional difficulties (SDQ-CR, range 0–40)	10.1 (6.2)	9.9 (5.9)	0.578	10.8 (5.5)	11.5 (6.3)	0.131
Emotional difficulties (SDQ-SR, range 0–40)	10.6 (5.6)	9.4 (5.4)	0.075	10.2 (5.6)	11.3 (6.2)	0.170
Diarrhea/Fever/Cough in last 2 weeks	199 (20.0%)	64 (17.9%)	0.375	508 (39.8%)	88 (46.8%)	0.067
N (%)^3^	993 (73.2%)	363 (26.8%)		1290 (87.2%)	190 (12.8%)	

1 Means (standard deviations; sd) and frequencies (percentages; %) at baseline among those retained at 36 months.

2 Means (standard deviations; sd) and frequencies (percentages; %) at baseline among those lost to follow-up at 36 months.

3 Frequencies (percentages; %) of institution-based and family-based children, respectively.

In order to describe the proportion of total variation in outcomes that was attributable to each of the three levels of the survey design (study sites, sampling units within sites, and individuals within sampling units), we fit linear mixed effects models for each continuous wellbeing measure, and changes in wellbeing over time, adjusting for age and gender and including random intercepts for sites and sampling units nested within sites. To further describe the proportion of variability in outcomes, after adjustment for study site, age, and gender that was attributable to overall differences between institutional and community-based care settings, we fit a second set of models that added fixed and random effects for a dichotomous variable indicating care setting type [36]. Analyses were conducted using Stata v.13.1 [37].

## Results

In total, 2,283 of the original 2,836 youth, or 80.5%, completed the 36-month assessment (Table 1). Of these, 1,290 (56.5%) were family-based and 993 (43.5%) resided in institutions. Approximately 73% of institution-based youth were retained at 36 months compared to 87% of family-dwelling youth. Of the 1,480 children enrolled from family settings, 45% were cared for by a primary caregiver other than the biological parent. Females were 47% of the family-living and 43% of the institution-based sample [5]. The median age was 9 years at baseline (range 6–12) and 12 years at the 3-year follow-up (range 8–16) [5]. In both family settings and institutions, children lost to follow-up were more likely to be older; however, those lost to follow-up did not differ by child sex or any wellbeing measure compared to those retained in the study at 36 months (Table 1).

**Figure 1:**
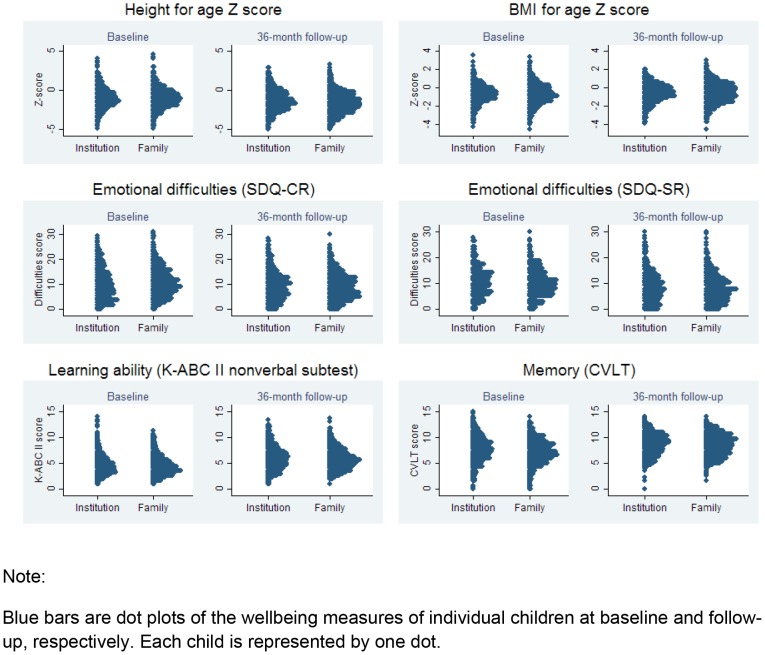
Distribution of child wellbeing measures at baseline and follow-up, by care setting type (Institution-based vs. family-based). Blue bars are dot plots of the wellbeing measures of individual children at baseline and follow-up, respectively. Each child is represented by one dot.

The distribution of the six continuous measures of health (height-for-age and BMI-for-age Z-scores), emotional wellbeing (SDQ-caregiver and self-reports), and cognition (K-ABC and CVLT-C) at baseline and 36 months, for the institution- and family-based samples, is shown in Figure 1. The figure shows substantial variability in all wellbeing measures within both institution- and family-based children, but similar distributions of these measures are seen across the institution- and family-based samples both at baseline and follow-up.

Table 2 presents the means for all dependent variables of interest and tests of the statistical significance of differences in means between family- and institution-based children at baseline and at the 36-month follow-up. For baseline, follow-up, and change measures, the magnitude of the differences between the institution- and family-based groups was small, especially when compared to the variation within each of these groups (See Figure 1). As seen in Panel A, at baseline, children in the institutional settings were doing statistically significantly better than children living in families on measures of general health, body-mass index, illness in the two weeks prior to the interview, learning ability, memory and caregiver-reported emotional wellbeing. Panel B presents the change over time between those in the institutions versus those living in family-based settings. This panel shows that over the 3 years, children in both institution-based and family-based settings improved on average on each measure except the height-for-age Z-score, which decreased in both groups. Children in family-based care improved statistically significantly more than those in institutions in general health, body-mass index and illness in the two weeks prior to the interview, memory, and caregiver-reported emotional wellbeing. Institution-based children had significantly less decline in height-for-age Z-scores than family-based children. At the 36-month follow-up, Panel C of Table 2 indicates that the institution-based children had statistically significantly higher means on measures of general health and height-for-age Z-scores and family-based children were rated by the caregivers as having significantly fewer emotional difficulties.

**Table 2:** Child outcomes in institution and community-based care settings at baseline and after 36 months.

					Weighted difference in	
	N		Unweighted distributions: Mean (SD) or N (%)		means or proportions, confidence interval, and p-value^3^	
	Institution-based	Family-based	Institution-based	Family-based	Institution vs. family	
**Panel A. Baseline** 1						
** Positive outcomes (higher score is better)**						
Caregiver-rated child health (1 to 5)	957	1263	4.0 (0.7)	3.7 (0.8)	0.38 (0.30;0.45)	<0.001
Height for age Z score (1 unit = 1 SD))	978	1280	−1.0 (1.4)	−1.0 (1.3)	−0.09 (−0.19;0.01)	0.076
BMI for age Z score (1 unit = 1 SD)	975	1280	−0.7 (1.0)	−0.7 (1.2)	0.10 (0.03;0.18)	0.009
Learning ability (K-ABC II, range 0–20))	984	1285	4.7 (1.9)	4.5 (1.8)	0.19 (0.04;0.34)	0.013
Memory CVLT, range 0–15))	970	1269	7.7 (2.3)	7.2 (2.2)	0.45 (0.23;0.67)	<0.001
** Negative outcomes (higher score or percentage is worse)**						
Emotional difficulties (SDQ-CR, range 0–40)	893	1211	10.0 (6.2)	10.8 (5.5)	−0.60 (−1.08;−0.12)	0.014
Emotional difficulties (SDQ-SR, range 0–40)	228	326	10.5 (5.5)	10.2 (5.6)	0.03 (−0.39;0.46)	0.874
Diarrhea/Fever/Cough in last 2 weeks	958	1269	194 (20.2%)	505 (39.8%)	−21% (−24%;−17%)	<0.001
**Panel B. Difference between baseline and follow-up** 2						
** Positive outcomes (higher score is better)**						
Caregiver-rated child health (1 to 5)	957	1263	0.2 (0.9)	0.5 (1.1)	−0.30 (−0.39;−0.22)	<0.001
Height for age Z score (1 unit = 1 SD))	978	1280	−0.4 (1.0)	−0.6 (1.0)	0.23 (0.16;0.31)	<0.001
BMI for age Z score (1 unit = 1 SD)	975	1280	0.1 (0.9)	0.2 (1.0)	−0.10 (−0.18;−0.03)	0.005
Learning ability (K-ABC II, range 0–20))	984	1285	1.3 (2.2)	1.2 (2.2)	−0.09 (−0.24;0.06)	0.244
Memory CVLT, range 0–15))	970	1269	0.9 (2.8)	1.2 (2.7)	−0.34 (−0.57;−0.12)	0.003
**Negative outcomes (higher score or percentage is worse)**						
Emotional difficulties (SDQ-CR, range 0–40)	893	1211	−0.7 (8.4)	−1.7 (7.3)	1.15 (0.52;1.77)	<0.001
Emotional difficulties (SDQ-SR, range 0–40)	228	326	−1.4 (7.7)	−1.8 (7.5)	0.28 (−0.34;0.90)	0.377
Diarrhea/Fever/Cough in last 2 weeks	958	1269	−18 (−1.9%)	−304 (−24.0%)	21% (17%;26%)	<0.001
**Panel C. 36-month follow-up**						
** Positive outcomes (higher score is better)**						
Caregiver-rated child health (1 to 5)	957	1263	4.2 (0.6)	4.2 (0.7)	0.07 (0.02;0.12)	0.005
Height for age Z score (1 unit = 1 SD)	978	1280	−1.4 (1.3)	−1.6 (1.3)	0.14 (0.05;0.23)	0.002
BMI for age Z score (1 unit = 1 SD)	975	1280	−0.6 (1.0)	−0.5 (1.1)	0.00 (−0.08;0.08)	0.981
Learning ability (K-ABC II, range 0–20)	984	1285	5.9 (2.1)	5.7 (1.9)	0.10 (−0.08;0.27)	0.265
Memory (CVLT, range 0–15)	970	1269	8.6 (2.1)	8.5 (2.1)	0.11 (−0.04;0.26)	0.160
**Negative outcomes (higher score or percentage is worse)**						
Emotional difficulties (SDQ-CR, range 0–40)	893	1211	9.3 (5.5)	9.1 (5.1)	0.55 (0.12;0.97)	0.011
Emotional difficulties (SDQ-SR, range 0–40)	228	326	9.1 (5.3)	8.5 (5.1)	0.31 (−0.18;0.80)	0.209
Diarrhea/Fever/Cough in last 2 weeks	958	1269	176(18.4%)	201 (15.8%)	1% (−2%;3%)	0.542

1 Baseline values include only those children who were not later lost to follow-up.

2 Difference represents child-level changes between baseline and follow-up.

3 Weighted difference accounts for age and sex differences in the distribution of children across study sites and differential rates of attrition between baseline and follow-up.

CVLT: California Verbal Learning Test. SDQ-CG: Strengths and Difficulties Questionnaire, Caregiver Report. SDQ-SR: SDQ, Self Report.

**Figure 2:**
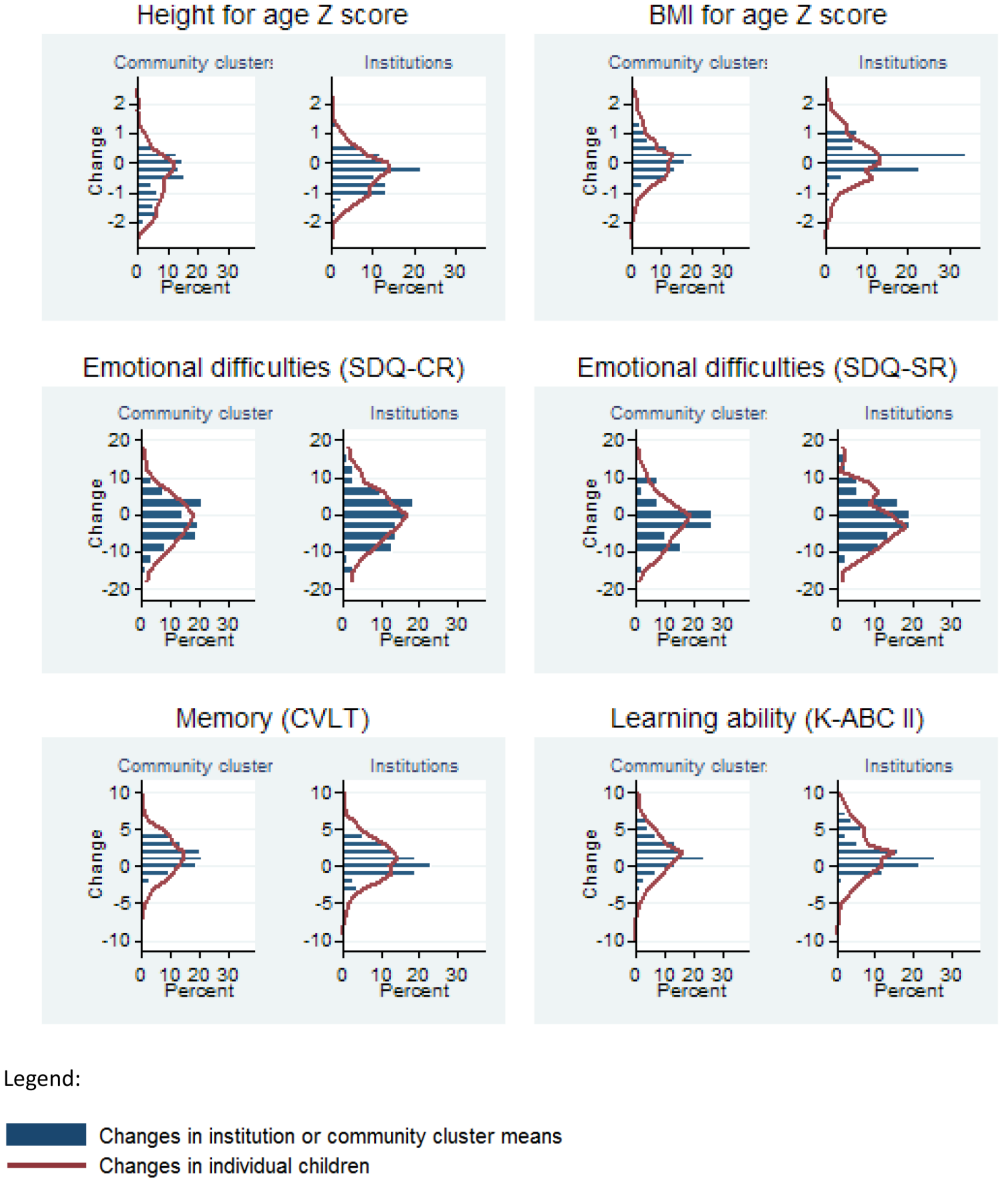
Changes in wellbeing of orphaned and separated children over 3 years, at the individual level and the institution or community cluster level. Sample size: Height for age – 252 community clusters, 1231 children; 73 institutions, 916 children. BMI for age – 247 community clusters, 1215 children; 73 institutions, 928 children. Total difficulties score, caregiver report – 245 community clusters, 1210 children; 69 institutions, 866 children. Total difficulties score, self-report – 38 community clusters, 326 children; 36 institutions, 221 children. Cognition (K-ABC II) – 256 community clusters, 1284 children; 73 institutions, 958 children. California Verbal Learning Test – 253 community clusters, 1268 children; 73 institutions, 944 children. The number of observations for self-reported total difficulties score is lower because only children at least 11 years old were asked for self-report. Numbers of observations vary across outcomes because of missing baseline or follow-up data (children) and because mean changes were only calculated for community clusters and institutions with at least 3 children with a change measure.

Figure 2 describes changes in wellbeing measures from baseline to 36 months both at the individual child level (solid line) and the sampling unit level (bars). Similar to Figure 1, substantial variability in changes is evident across children and across institutions and community clusters, while little difference is evident in the overall distributions when comparing institution-based and family-based children.

Table 3 describes the proportion of overall variation in wellbeing that is explained by differences between the six sites, between sampling units within sites, and between children within settings, and the proportion of overall variation that is explained by comparing institution-based children as a whole to family-based children as a whole. The first 3 columns represent how much of the variation in outcomes is attributable to the six study sites (column 1), the 394 sampling units (83 institutions and 311 community clusters) (column 2), and differences between children within each sampling unit (column 3), after controlling for age and gender. Note that for row the percentages across these three columns add to 100%. The fourth column represents the percent of variation in wellbeing measures explained by a dichotomous variable for caregiving type (i.e. institution vs. family-based) after controlling for age, gender and study site. At baseline the percent of variation in wellbeing attributable to differences between sites ranged from 2.8–27.7%, depending on the wellbeing measure, while the percent attributable to differences between sampling units within sites ranged from 6.5–21.3% and the percent attributable to differences between individual children within sampling units ranged from 61.2–85.6% (Panel A). Similar patterns were observed for variation in outcomes at the 36-month follow-up (11.4–27% explained at the site level; 7.9–14% explained at the sampling unit level; 60–74.6% explained at the individual level; Panel C), and for variation in the change in outcomes between baseline and 36 months (between 7.1–37.1% was explained at the site-level; 7.9–16.4% explained at the level of sampling unit; 55–78.6% explained at the individual level; Panel B). Importantly, the percent of variation in outcomes explained by the dichotomization of care setting type, institution- versus family-based care, ranged from 0.1–7.3% at baseline, from 0.1–2.7% at 36-month follow-up, and from 0.1–4% for changes in outcomes between baseline and 36 months.

**Table 3:** Variation in wellbeing attributable to the site, setting, and individual levels.

	Percent of variation attributable to differences between:^1^			
	Sites	Sampling Units^2^ within sites	Individuals within care settings	Percent of variation in outcome changes explained by care setting type^3^
**Panel A. Variation at baseline**				
Caregiver-rated child Health	6.4%	21.3%	72.3%	4.0%
Height for age Z score	8.0%	6.5%	85.5%	0.5%
BMI for age Z score	15.2%	12.6%	72.3%	7.3%
Emotional difficulties (SDQ-SR/CG)	26.1%	12.8%	61.2%	0.1%
Emotional difficulties (SDQ-CR)	20.4%	17.0%	62.5%	0.5%
Emotional difficulties (SDQ-SR)	27.7%	8.8%	63.6%	1.2%
Learning ability (K-ABC II nonverbal subtest)	5.7%	12.5%	81.7%	1.5%
Memory (CVLT)	2.8%	11.6%	85.6%	2.9%
**Panel B. Variation in changes between baseline and follow-up**				
Caregiver-rated child Health	20.3%	13.2%	66.4%	1.7%
Height for age Z score	37.1%	7.9%	55.0%	4.0%
BMI for age Z score	7.1%	14.2%	78.6%	1.0%
Emotional difficulties (SDQ-SR/CG)	25.5%	11.3%	63.3%	0.1%
Emotional difficulties (SDQ-CR)	25.0%	12.9%	62.1%	1.6%
Emotional difficulties (SDQ-SR)	27.1%	16.4%	56.5%	0.1%
Learning ability (K-ABC II nonverbal subtest)	27.0%	11.5%	61.5%	0.0%
Memory (CVLT)	24.4%	13.0%	62.6%	0.1%
**Panel C. Variation at follow-up**				
Caregiver-rated child Health	18.4%	13.1%	68.4%	1.7%
Height for age Z score	23.5%	7.9%	68.6%	1.5%
BMI for age Z score	11.4%	14.0%	74.6%	2.7%
Emotional difficulties (SDQ-SR/CG)	26.6%	10.8%	62.6%	0.2%
Emotional difficulties (SDQ-CR)	24.5%	12.1%	63.4%	1.0%
Emotional difficulties (SDQ-SR)	26.1%	10.0%	63.9%	0.1%
Learning ability (K-ABC II nonverbal subtest)	27.0%	12.9%	60.0%	0.3%
Memory (CVLT)	24.3%	11.6%	64.1%	0.5%

1 From a linear mixed model adjusted for age and gender and including random effects for sites and care settings.

2 Institutions or community clusters sampled within sites.

3 Percent reduction in overall variance upon introduction of dichotomous variable and random site-level slopes for setting type, conditional on site, age, and gender.

## Discussion

The POFO study is one of the few studies to look at a longitudinal cohort of OSC who are over the age of 5 and growing up in a variety of LMIC sites and settings. The analyses presented in this manuscript demonstrate substantial individual-level variation across eight measures of health and wellbeing over three years among OSC living in institutions and OSC living with families. Some children are doing relatively well and others relatively poorly across all measures and over time. At the aggregate, children in both settings improved on most wellbeing measures over time, while the family-based children improved more over time, and could be catching up to the wellbeing status of the institution-based children. This overall trend in improvement and the potential for increased improvement across groups on various well-being measures is an encouraging finding. However, it is important to note that in nearly all cases the magnitude of both the differences between institution- and family-based children and the differences in changes over time were substantively negligible when compared to the variation between children within settings. There are relatively few systematic differences evident when comparing average health and wellbeing between institution-based and family-based OSC as a whole. Further, residential setting does not seem to meaningfully account for either positive or negative change over time. In other words, there are substantial numbers of children in institutions and family settings doing relatively well and poorly both cross-sectionally and over time, which underscores the need to decipher the microcosm of quality of care issues within each setting.

The findings presented here complement the recent work of other researchers who have included community comparison groups when examining the wellbeing of children in group homes. While multiple studies from LMICs examined child wellbeing in institutions without comparison groups [38]–[40], there are a growing number of studies with rigorous research designs that are attempting to tease apart OSC wellbeing as it relates to family-based and institution-based care settings. These researchers have found that nutritional status of children in institutions in Kenya [19] and psychosocial status of children in group homes in China [20] was somewhat better than for those living with families. The Kenyan study also found that children in group homes were more likely to have their basic needs met [7]. Many studies over the years have documented the difficult lives that orphans face living in family-based settings including poverty, stigma, lack of educational resources, and exposure to physical and sexual violence [41]–[43]. Still other studies caution that returning children from institutions to biological families may not result in the best outcomes, at least without significant intervention with the biological family, including supervision and follow-up. While it is widely cited that a significant proportion of children living in group homes have biological parents, it has also been found that up to 90% of those with a biological parent were being maltreated before entering the institution [41]. A study conducted in Russia found that infants in socially and emotionally deprived institutions compared poorly to those living in families, but that over time, children who were reunified with their original biological families from the institutions fared worse than those who remained in the institutional settings [9]. Studies from both Romania and Russia have also shown that improvements can be made to institutional care settings that result in more positive outcomes for children [10], [12]. These studies and ours should not be interpreted to mean that institutions are the preferred living environment for children, but rather that a family-based setting is not guaranteed to be a better place for a child to live. It is likely that the quality of care provided within a setting, whether that setting be family-based or institution-based, makes the difference in child wellbeing outcomes. The oft-cited Bucharest study in Romania, for example, found that when infants from emotionally and socially deprived institutions were assigned to live with government foster care families, they did not improve nearly as well as the children who had previously been assigned to well trained, paid and supervised foster care families, demonstrating that it was care quality, not care setting, accounting for differences [44].

Residential institutions represent a wide range of child care models with diverse resources, cultural traditions, and risk/resilience environments that can serve as protective factors in the face of extreme hardship. This analysis and others comparing children cared for in institutions and community settings suggest that institutional living may not inevitably result in damage during childhood, especially when put in the context of families and communities that are themselves resource-challenged. In order to improve child outcomes, children must transition from the lower to the upper ranges of the health and wellbeing outcome distributions. Since institutional residence, broadly defined, does not appear to account for poorer outcomes among OSC, it is important to understand the caregiving characteristics, both in group-based and family-based settings, that are associated with more positive outcomes. With a large and growing number of children living on the streets in many LMICs [45], there is a critical need not only for additional care setting options but also those to be supported to provide high quality care.

Important strengths of this analysis include the inclusion of six culturally diverse sites from 5 LMICs, the carefully selected comparison group, the rigorous sampling methodology that yielded statistically representative cohorts of institution-based and family-based OSC from the same communities, the longitudinal study design, the high retention, and the wide variety of carefully measured health, cognition, and emotional development wellbeing measures. Several limitations should also be considered. First, although data for POFO were collected across six culturally, politically, religiously, and geographically distinct LMIC sites, corresponding to a sizable portion of the orphaned and separated children worldwide, there is no representation from South America or Eastern Europe, where earlier research on institutional care originated. Recognizing that contexts are not necessarily interchangeable, continued study and inclusion of other contexts and conditions are needed to better understand the various trajectories of positive and negative outcomes in different settings. Second, given the observational design of the study, control for natural events and child and caregiver characteristics prior to entry into the study through randomization is not possible. The longitudinal design, which in effect allows each child to serve as his or her own control, enhances the validity of our conclusions about the relative wellbeing of institution-living and family-living OSC. Finally, although we were able to compare the overall wellbeing of institution-living and family-living OSC, the present analysis was not able to explore specific caregiving characteristics that may influence child outcomes in both institutional and family settings, such as emotional attachment with the caregiver. Further research is needed to identify risk and protective factors in both institutional and family settings.

Taken as a whole, the study findings do not support the hypothesis that institution-based living universally and significantly adversely affects child wellbeing. Given that the greatest variation in wellbeing exists within the care settings, whether institutions or families, the way to improve wellbeing in the greatest number of OSC would be to implement improvements within each care setting type so that those on the lower range of the wellbeing curves move to the middle and upper ranges. This should best be accomplished by understanding which caregiving characteristics within a setting are associated with more positive outcomes.

## References

[1] The World Bank Group (2013) How we Classify Countries. Available: http://data.worldbank.org/about/country-classifications. Accessed 2013 Dec 5.

[2] UNICEF (2008) Orphans. Available: http://www.unicef.org/media/media_45279.html. Accessed 2013 Dec 5.

[3] UNICEF (2006) The State of the World’s Children 2006: Excluded and Invisible. Available: http://www.unicef.org/sowc06/pdfs/sowc06_fullreport.pdf. Accessed 2013 Oct 1.

[4] UNICEF (2009) Progress for Children A Report Card on Child Protection. Available: http://www.unicef.org/publications/files/Progress_for_Children-No.8_EN_081309.pdf. Accessed 2013 Dec 5.

[5] Whetten K, Ostermann J, Whetten RA, Pence BW, O’Donnell K, et al (2009) A Comparison of the Wellbeing of Orphans and Abandoned Children Ages 6–12 in Institutional and Community-Based Care Settings in 5 Less Wealthy Nations. PLoS One 4: e8169 10.1371/journal.pone.0008169 20020037 PMC2790618

[6] NGO Working Group (2013) Identifying Basic Characteristics of Formal Alternative Care Settings For Children. Available: http://www.fice-inter.net/wp-content/uploads/2013/04/Formal_care_settings_characteristics_March_2013_final.pdf. Accessed 2013 Nov 11.

[7] Embleton L, Ayuku D, Kamanda A, Atwoli L, Ayaya S, et al (2014) Models of care for orphaned and separated children and upholding children’s rights: cross-sectional evidence from western Kenya. BMC International Health and Human Rights 14: 9 DOI: 10.1186/1472-698X-14-9. Available: 10.1186/1472-698X-14-9 http://www.biomedcentral.com/1472-698X/14/9 Available: http://www.biomedcentral.com/1472-698X/14/9 Accessed 2014 April 1 24685118 PMC4021203

[8] Van IJzendoorn MH, Luijk MPCM, Juffer F (2008) IQ of children growing up in children's homes: A meta-analysis on IQ delays in orphanages. Merrill-Palmer Quarterly 54: 341–356.

[9] Merz EC, McCall RB, Groza V (2013) Parent-Reported Executive Functioning in Postinstitutionalized Children: A Follow-Up Study. Journal of Clinical Child & Adolescent Psychology 42: 726–733 10.1080/15374416.2013.764826 23413815 PMC3660422

[10] McCall RB, Groark CJ, Fish L, Muhamedrahimov RJ, Palmov OI, et al (2013) Maintaining a social-emotional intervention and its benefits for institutionalized children. Child Development 84: 734–49 10.1111/cdev.12098 PMC3706532 23551051

[11] Zeanah CH, Nelson CA, Fox NA, Smyke AT, Marshall P, et al (2003) Designing research to study the effects of institutionalization on brain and behavioral development: The Bucharest Early Intervention Project. Dev Psychopathol 15: 885–907 10.1017/S0954579403000452 14984131

[12] Nelson CA 3rd, Zeanah CH, Fox NA, Marshall PJ, Smyke AT, et al.. (2007) Cognitive recovery in socially deprived young children: the Bucharest Early Intervention Project. Science 318: 1937–40. DOI: 10.1126/science.1143921. 10.1126/science.1143921 18096809

[13] United States Government Action Plan on Children in Adversity: A Framework for International Assistance: 2012–2017 (2012) Available: http://www.dol.gov/ilab/programs/ocft/ChildrenInAdversityPlan.pdf. Accessed 2013 Dec 5.

[14] th Congress, 2013–2015 (2013) H.R.3323: Children in Families First Act of 2013. Available: https://www.govtrack.us/congress/bills/113/hr3323/text. Accessed: 2013 Dec 5.

[15] Johnson DE, Dole K (1999) International adoption: Implications for early intervention. Infants & Young Children 11: 34–45.

[16] Aboud F, Samuel M, Hadera A, Addus A (1991) Intellectual, social and nutritional status of children in an Ethiopian orphanage. Soc Sci Med 33: 1275–1280 10.1016/0277-9536(91)90075-N 1776040

[17] Otieno PA, Nduati RW, Musoke RN, Wasunna AO (1999) Growth and development of abandoned babies in institutional care in Nairobi. S Afr Med J 76: 430–5. 10520347

[18] Wolff PH, Tesfai B, Egasso H, Aradom T (1995) The orphans of Eritrea: A comparison study. J Child Psychol Psychiatry 36: 633–44 10.1111/j.1469-7610.1995.tb02318.x 7650087

[19] Braitstein P, Ayaya S, Nyandiko WM, Kamanda A, Koech J, et al (2013) Nutritional Status of Orphaned and Separated Children and Adolescents Living in Community and Institutional Environments in Uasin Gishu County, Kenya. PLoS ONE 8: e70054 10.1371/journal.pone.0070054 23922900 PMC3724723

[20] Hong Y, Li X, Fang X, Zhao G, Zhao J, et al (2011) Care arrangements of AIDS orphans and their relationship with children’s psychosocial well-being in rural China. Health Policy Plan 26: 115–123 10.1093/heapol/czq025 20587602 PMC3040369

[21] O'Donnell K, Murphy R, Ostermann J, Masnick M, Whetten RA, et al (2012) A brief assessment of learning for orphaned and abandoned Children in low and middle-income countries. AIDS Behav 16: 480–90 10.1007/s10461-011-9940-z 21538088 PMC3817622

[22] Whetten KJ, Ostermann RA, Whetten K, O’Donnell NM, Thielman NM, et al (2011) More than the loss of a parent: Potentially Traumatic Events among Orphaned and Abandoned Children. J Trauma Stress 24: 174–182 10.1002/jts.20625 21442663 PMC3610328

[23] Thielman N, Ostermann J, Whetten K, Whetten R, O'Donnell K (2012) Correlates of Poor Health among Orphans and Abandoned Children in Less Wealthy Countries: The Importance of Caregiver Health. PLoS One 7: e38109 10.1371/journal.pone.0038109 22719867 PMC3374817

[24] Messer L, Pence BW, Whetten K, Whetten RA, O’Donnell K, et al (2010) HIV-stigma and attributes of institutional- and community-based caregivers of orphans and vulnerable children living in five less-wealthy countries. BMC Public Health 10: 504 10.1186/1471-2458-10-504 20723246 PMC2936424

[25] Ware J, Kosinski M (2004) SF-36 Physical and Mental Health Summary Scales: A Manual for Users of Version 1. 2nd ed. Lincoln, RI: Quality Metric, Inc. 238 p.

[26] World Health Organization (2006) WHO Child Growth Standards: Length/height-for-age, weight-for-age, weight-for-length, weight-for-height and body mass index-for-age. Available: http://www.who.int/childgrowth/standards/Technical_report.pdf. Accessed 2013 Dec 5.

[27] Goodman R (1997) The Strengths and Difficulties Questionnaire: a research note. J Child Psychol Psychiatry 38: 581–6 10.1111/j.1469-7610.1997.tb01545.x 9255702

[28] Goodman R, Meltzer H, Bailey V (1998) The Strengths and Difficulties Questionnaire: A pilot study on the validity of the self-report version. Eur Child Adolesc Psychiatry 7: 125–130 10.1080/0954026021000046137 9826298

[29] Goodman R (2001) Psychometric properties of the strengths and difficulties questionnaire. J Am Acad Child Adolesc Psychiatry 40: 1337–1345 10.1097/00004583-200111000-00015 11699809

[30] Syed EU, Hussein SA, Azam SI, Khan AG (2009) Comparison of Urdu version of Strengths and Difficulties Questionnaire (SDQ) and the Child Behaviour Check List (CBCL) amongst primary school children in Karachi. J Coll Physicians Surg Pak 19: 375–9 DOI: 06.2009/JCPSP.375379 19486578

[31] Muris P, Maas A (2004) Strengths and difficulties as correlates of attachment style in institutionalized and non-institutionalized children with below-average intellectual abilities. Child Psychiatry and Human Development 34: 317–328 10.1023/B:CHUD.0000020682.55697.4f 15039604

[32] Richter J, Sagatun A, Heyerdahl S, Oppedal B, Roysamb E (2011) The Strengths and Difficulties Questionnaire (SDQ) - self-report. An analysis of its structure in a multiethnic urban adolescent sample. Journal of Child Psychology and Psychiatry 52: 1002–11 10.1111/j.1469-7610.2011.02372.x 21418061

[33] Kaufman AS, Kaufman NL (2004) Kaufman assessment battery for children (KABC-II). 2nd ed. Circle Pines, MN: Guidance Services Publishing.

[34] Skuy M, Taylor M, O'Carroll S, Fridjhon P, Rosenthal L (2000) Performance of black and white South African children on the Wechsler Intelligence Scale for Children–Revised and the Kaufman Assessment Battery. Psychol Rep 86: 727–737 10.2466/pr0.2000.86.3.727 10876320

[35] Delis D, Kramer J, Kaplan E, Ober B (1987) California Verbal Learning Test (CVLT). San Antonio, TX: Psychological Corporation.

[36] Rabe-Hesketh S, Skrondal A, Pickles A (2005) Maximum likelihood estimation of limited and discrete dependent variable models with nested random effects. J Econom 128: 301–323 10.1016/j.jeconom.2004.08.017

[37] StataCorp (2013) Stata Statistical Software: Release 13. College Station, TX: StataCorp LP.

[38] Powell G (2006) Children in institutional care: lessons from Zimbabwe's experience. Journal of Social Development in Africa 21: 130–146.

[39] Wanat S, Whisnant J, Reicherter D, Solvason B, Juul S, et al (2010) Coping with the challenges of living in an Indonesian residential institution. Health Policy 96: 45–50 10.1016/j.healthpol.2010.01.001 20102784

[40] Morantz G, Heymann J (2008) Life in institutional care: the voices of children in a residential facility in Botswana. AIDS Care 22: 10–16 10.1080/0954012090301260 20390476

[41] Morantz G, Cole DC, Ayaya S, Ayuku D, Braitstein P 10 01 (2013) Maltreatment experiences and associated factors prior to admission to residential care: A sample of institutionalized children and youth in western Kenya. Child Abuse & Neglect 37: 778–787 10.1016/j.chiabu.2012.10.007 23290620 PMC3633719

[42] Cluver L, Orkin M (2009) Cumulative risk and AIDS-orphanhood: Interactions of stigma, bullying and poverty on child mental health in South Africa. Social Science & Medicine 69: 1186–1193 10.1016/j.socscimed.2009.07.033 19713022

[43] Cluver LD, Orkin M, Gardner F, Boyes ME (2012) Persisting mental health problems among AIDS-orphaned children in South Africa. Journal of Child Psychology and Psychiatry, and Allied Disciplines 53 (4): 363–70 10.1111/j.1469-7610.2011.02459.x 21883206

[44] McLaughlin KA, Zeanah CH, Fox NA, Nelson CA (2012) Attachment security as a mechanism linking foster care placement to improved mental health outcomes in previously institutionalized children. Journal of Child Psychology and Psychiatry 53: 46–55 10.1111/j.1469-7610.2011.02437.x 21733136 PMC3193871

[45] Moccia P, Anthony D (2005) The state of the world's children 2006: Excluded and invisible. New York: UNICEF. Available: http://www.unicef.org/sowc06/pdfs/sowc06_fullreport.pdf. Accessed: 2014 June 16.

